# Measuring Post-transfusion Recovery and Survival of Red Blood Cells: Strengths and Weaknesses of Chromium-51 Labeling and Alternative Methods

**DOI:** 10.3389/fmed.2018.00130

**Published:** 2018-05-15

**Authors:** Camille Roussel, Pierre A. Buffet, Pascal Amireault

**Affiliations:** ^1^Biologie Intégrée du Globule Rouge UMR_S1134, INSERM, Univ. Paris Diderot, Sorbonne Paris Cité, Univ. de la Réunion, Univ. des Antilles, Paris, France; ^2^Institut National de la Transfusion Sanguine, Paris, France; ^3^Laboratoire d’Excellence GR-Ex, Paris, France; ^4^Laboratory of Cellular and Molecular Mechanisms of Hematological Disorders and Therapeutic Implications U1163/CNRS ERL 8254, INSERM, CNRS, Univ Paris Descartes, Sorbonne Paris Cité, Paris, France; ^5^Université Paris Descartes, Paris, France; ^6^Assistance publique des hôpitaux de Paris, Paris, France

**Keywords:** transfusion recovery, red blood cell, spleen, red blood cell morphology, red blood cell deformability, storage lesion

## Abstract

The proportion of transfused red blood cells (RBCs) that remain in circulation is an important surrogate marker of transfusion efficacy and contributes to predict the potential benefit of a transfusion process. Over the last 50 years, most of the transfusion recovery data were generated by chromium-51 (^51^Cr)-labeling studies and were predominantly performed to validate new storage systems and new processes to prepare RBC concentrates. As a consequence, our understanding of transfusion efficacy is strongly dependent on the strengths and weaknesses of ^51^Cr labeling in particular. Other methods such as antigen mismatch or biotin-based labeling can bring relevant information, for example, on the long-term survival of transfused RBC. These radioactivity-free methods can be used in patients including from vulnerable groups. We provide an overview of the methods used to measure transfusion recovery in humans, compare their strengths and weaknesses, and discuss their potential limitations. Also, based on our understanding of the spleen-specific filtration of damaged RBC and historical transfusion recovery data, we propose that RBC deformability and morphology are storage lesion markers that could become useful predictors of transfusion recovery. Transfusion recovery can and should be accurately explored by more than one method. Technical optimization and clarification of concepts is still needed in this important field of transfusion and physiology.

## Introduction

Each year, more than 85 million red blood cells (RBCs) units are transfused worldwide. This demanding human and organizational task is conducted by national or local organizations. Collection, transformation, storage (for a maximum of 35–49 days), and distribution of blood products are tightly quality controlled, most commonly at the national level. In industrialized countries, most of the transfused RBCs are stored as red cell concentrates (RCC), from which plasma, platelets, and leukocytes have been almost entirely removed, usually using centrifugation and/or leukoreduction filters.

The objective of an RCC transfusion is to increase the oxygenation capacity of the recipient by increasing the number of functional RBC in circulation. Improvement in tissue oxygenation following transfusion is arguably the most relevant marker of transfusion efficacy but measuring it is technically and logistically challenging in patients and impossible in healthy volunteers in whom tissue oxygenation is not altered. Measuring the proportion of RBCs that remain in circulation after transfusion thus appears as a suitable surrogate marker to evaluate the efficacy of a transfusion. That a reasonable proportion of transfused RBC stays in circulation for long enough to operate the expected correction is indeed a prerequisite for transfusion efficacy.

Early studies have identified that, after storage, a variable proportion of transfused RBC is removed from the circulation in the first 24 h following transfusion (1). Then, the remaining transfused RBCs have a normal survival. Although the long-term survival of RBC is an important parameter to evaluate transfusion efficacy, most studies have focused on the measure of the 24 h transfusion recovery.

Several techniques have been developed and used in the last 100 years to measure transfusion recovery. Transfusion recovery using chromium-51 (^51^Cr) labeling is now a regulation criterion to license new storage systems or RCC preparation processes by the Food and Drug Administration (FDA). The FDA threshold to approve a preparation and storage process of RBC is a maximum 1% *in vitro* hemolysis and a 24 h *in vivo* recovery of at least 75% after reinfusion of autologous ^51^Cr-labeled RBC in healthy volunteers, at the limit of storage (2). The ^51^Cr-labeling technique was first used in the early 1950s and became the gold standard in the 1970s when the International Committee for Standardization in Hematology (3) proposed it as the reference technique. The use of a standardized protocol is essential to compare studies distant in space or time. Over the last 50 years, most of the transfusion recovery data were generated by ^51^Cr-labeling studies, mostly to validate new storage systems and RCC preparation processes. As a consequence, our understanding of transfusion efficacy strongly depends on the strengths and weaknesses of ^51^Cr labeling. However, other methods have been developed and validated, the advantages and limitations of which deserve careful analysis.

We will provide a brief overview of the methods used to measure transfusion recovery in humans. We will compare their strengths and weaknesses and critically analyze their potential limitations. Also, based on our understanding of the spleen-specific filtration of damaged RBC, we will discuss the relevance of storage lesion markers to predict transfusion recovery. We will finally discuss the current state of knowledge in the RBC transfusion field and propose future directions. Animal studies published in the recent years on this topic are beyond the scope of this analysis.

## Methods Used for Transfusion Recovery Studies

### Differential Agglutination (DA)

Differential agglutination was the first method used to measure transfusion recovery of a complete RCC (1, 4). In the 24 h post-transfusion blood sample, RBCs from the donor or the recipient are agglutinated with an appropriate antiserum, and the remaining RBCs are counted (5, 6). Similarly, an automated DA technique was developed where agglutinates are removed automatically and the remaining hemoglobin is quantified colorimetrically (7–13). The “100%” initial point is calculated from the prediction of the recipient’s normal blood volume (using its height and weight). Alternatively, the initial point can be obtained from an estimation of the recipient’s red cell volume using radioactive labeling of “fresh” RBC.

### Radioactive Labeling Methods With ^51^Cr and Other Isotopes

Here, 15–30 ml of RBCs from a donor is labeled with ^51^Cr (14) and injected to the recipient (most of the time the donor himself) (15–31). RBC recovery is quantified after transfusion by taking a blood sample at early time points (5, 7.5, 10, 12.5, and 15 min) and 24 h after injection. In each sample, a radioactivity count number is acquired, and the initial point is extrapolated by linear regression. Alternatively, transfusion recovery can be evaluated using the technetium-99 (^99m^Tc)/^51^Cr double-labeling technique (32–43). In this method, the recipient’s RBC volume is first evaluated using a known amount of “fresh” RBC labeled with ^99m^Tc (^32^P and ^52^Cr were also used in older studies) (44–46), which is then used to calculate transfusion recovery. Similarly, ^51^Cr labeling can be associated with ^125^I-labeled albumin to evaluate the recipient’s plasma volume, which is then used in the calculation of transfusion recovery (35, 46–48). A reduced transfusion recovery was observed in some studies (34, 46, 48) that compared the double labeling with the single-labeling method. This is probably due to an undervaluation of the very short-term component of survival when using the single-label method (since some RBCs are rapidly removed from the circulation in the very first minutes following injection) and suggests that the double-label method is worth the extra complexity.

### Biotinylation

Red blood cells from a donor (5–30 ml) are labeled with biotin and injected to the recipient. RBC recovery is usually quantified after transfusion by taking a blood sample at an early time point (10 min) and 24 h after injection (49, 50). Fluorescent labeling of biotinylated RBC and flow cytometry detection quantify the proportion of transfused RBC. By labeling RBC with different densities of biotin, it is possible to evaluate the recovery of up to three RBC populations in the same recipient. Care must be taken to avoid too high concentrations of biotin since it has been correlated with increase of transfused RBC clearance and anti-biotin antibodies in the recipient (51). A GMP grade biotin is now available and could be used in countries where radioactive labeling procedures are not authorized (52). This method theoretically allows the determination of transfused RBC characteristics.

### Minor Antigen Mismatch

In the minor antigen mismatch method, an antibody directed against a minor antigen (e.g., Fy), which differs between the donor and the recipient, is used to determine, by flow cytometry, the proportion of transfused RBC in the 24 h post-transfusion sample (53, 54). No manipulation of the transfused RBC is necessary, and recovery is evaluated after the transfusion of a complete RCC, but the measure is dependent on the prediction of the recipient blood volume (from its height and weight). Theoretically, the characteristics of transfused RBC can be observed after transfusion.

### Increase in Blood Counts

A simple method to evaluate transfusion recovery is to measure the increase in blood count (hemoglobin level or hematocrit) between a pretransfusion sample and post-transfusion samples (55–58). Limitations from such a method include the limited accuracy of the blood count measure and the unknown recipient’s blood volume (and its variable response to transfusion) that both contribute to the inaccuracy of the measure.

## Limitations of Existing Recovery Studies

Strengths and weaknesses of the different methods are summarized in Table 1. One of the potential bias of the chromium or biotinylation techniques stems from the labeling protocol necessary to perform these studies. In the normal transfusion setup, RBCs are transfused to the recipient directly from the bag while in these two methods, RBCs are manipulated, centrifuged, and incubated in PBS or saline solution. It is conceivable that these steps modify labeled RBC in a way that affects their ability to stay in circulation, although a recent study showed that some RBC properties are only slightly modified by the biotinylation protocol (59). The situation is different with DA and minor antigen mismatch, as these techniques do not require any RBC manipulation before transfusion, thereby eliminating this potential source of artifact.

**Table 1:** Strengths and weaknesses of the different methods to measure transfusion recovery.

Method	Principle	Strengths	Weaknesses
Differential agglutination (DA)	Red blood cells (RBCs) from the donor or recipient are agglutinated, and the remaining RBCs are counted	Transfusion recovery of a normal transfusion volume can be determined One or more RBC populations can be quantified in parallel The persistence of transfused RBC in circulation may be followed for several weeks The method can be used in patients, in infants, pregnant women, and any vulnerable group RBCs from the donor or the recipient are not manipulated before transfusion	Quantification is inaccurate when/if agglutination is incomplete Only allogeneic RBC transfusion can be studied with this method The method is dependent on the prediction of the recipient’s blood volume (from its height and weight) or the calculation of the recipient RBC volume using radioactivity
Automated DA	RBCs from the donor or recipient are agglutinated and the remaining hemoglobin is quantified	Variability is reduced when an automated procedure is used	

Chromium-51 (^51^Cr)	Donor RBCs are labeled with ^51^Cr and then injected to the recipient Recovery is quantified in serial samples	This is a reference Food and Drug Administration-approved method to test new devices/procedures for transfusion/storage The procedure is standardized which allows comparison between different studies Autologous RBC transfusion can be studied with this method	Only relatively small volumes (15–30 ml) of labeled RBC can be transfused Elution of ^51^Cr from RBC limits the evaluation of long-term persistence in circulation (less than 30 days) There are regulatory, logistical, and technical constraints related to the use of radioactivity Protected populations cannot be studied because recipients are exposed to radioactivity RBCs from the donor are manipulated before transfusion
Technetium-99 (^99m^Tc)/^51^Cr	Blood volume in the recipient is first evaluated using a known amount of tracer “fresh” RBC labeled with ^99m^Tc	Quantification is expected to be more robust because recipient’s RBC volume is measured with ^99m^Tc-labeled RBC	

Biotin	One or more donor RBC populations are labeled with different concentrations of biotin then quantified in serial samples by flow cytometry	The persistence of transfused RBC in circulation may be followed for several weeks Up to 3 RBC populations can be quantified in parallel Autologous RBC transfusion can be studied with this method The method can be used in patients, in infants, pregnant women, and any vulnerable group The characteristics of transfused RBC can be observed after transfusion	Only relatively small volumes (15–30 ml) of labeled RBC can be transfused The recipient is at risk of developing anti-biotin antibodies RBCs from the donor are manipulated before transfusion

Antigen mismatch	Following transfusion of compatible RBC, minor antigen differences (e.g., Fy) are used to quantify RBC from the donor by flow cytometry	Transfusion recovery of a normal transfusion volume can be determined The persistence of transfused RBC in circulation may be followed for several weeks One or more RBC populations can be quantified in parallel The characteristics of transfused RBC can be observed after transfusion The method can be used in patients, in infants, pregnant women, and any vulnerable group RBCs from the donor or the recipient are not manipulated before transfusion	Only compatible transfusions with at least 1 minor antigen difference can be studied with this method The method is dependent on the prediction of the recipient’s blood volume (from its height and weight)

Increase in blood counts	Blood hemoglobin levels (or hematocrit) are measured before and after transfusion	Transfusion recovery of a normal transfusion volume can be determined The method can be used in patients, in infants, pregnant women, and any vulnerable group RBCs from the donor or the recipient are not manipulated before transfusion	The quantification is inaccurate when the blood volume of the recipient is abnormal Processes or interventions other than transfusion can impact on hemoglobin blood level (or hematocrit)

Another potential limitation of the accuracy of the ^51^Cr or biotinylation techniques is the infusion of a relatively small volume (5–30 ml) of RBC rather than a complete RCC. Transfusion recovery may indeed be influenced by the volume of transfused RBC. To explore this possibility and mimic more closely a complete RCC transfusion, ^51^Cr-labeled or biotinylated RBC could be co-transfused with the rest of the RCC. One study (60) reported a lower transfusion recovery of an entire unit (using automated DA) when compared with a 10–30 ml transfusion volume (using ^51^Cr labeling). However, it is not possible to ascertain that the “volume of transfusion” was responsible for this difference since other potential factors related to the method used to quantify transfusion recovery method (automated DA vs ^51^Cr) may have impacted the observation. The exact clearance mechanism(s) of potentially damaged RBC stored for many weeks are not well known. To what extent transfusion recovery data using a small amount of RBC accurately predict the outcome of a complete—or massive—RCC transfusion remains therefore an open question.

To reduce the risk of adverse events including transmission of infectious diseases, most of the transfusion recovery studies are conducted using autologous transfusion of stored RBC to healthy volunteers. In this setup, conditions of transfusion are probably appropriate to evaluate storage and donor effects. However, they do not take into consideration the possible complex interaction between damaged-stored RBC and potential recipient specificities related to its physiopathological condition. Along this line, it has been shown that survival of transfused RBC is abnormally low in some thalassemia patients with splenomegaly (61). Normal survival was restored following splenectomy suggesting that the spleen is where most RBCs that were no longer present in the circulation 24 h after transfusion had been retained. This is an example of how the medical condition of the recipient can impact transfusion recovery.

## Storage Lesion, Transfusion Recovery, and Spleen Filtration

### Storage Lesion and Transfusion Recovery

Recently, a number of prospective clinical studies have been conducted to evaluate the potential benefit of transfusing RCC stored for a short period (61–66). These studies have shown that transfusion of RCC stored for a short period does not reduce in-hospital morbidity or mortality in adult and children transfused for acute anemia. The “standard of care” collection and storage processes thus appear to be currently adequate when (accurately) assessed on clinical endpoints. However, these complex prospective clinical studies assessing predominantly safety may be difficult to conduct in some cohorts, such as chronically transfused patients, where the long-term impact of transfusions may be even more relevant. In addition, clinical studies of safety did not specifically examine the effect of transfusing RCC stored for a long period (more than 35 days) and did not directly address the efficacy of the procedure. This evaluation could be important in light of the well-documented RBC alterations that accumulate during hypothermic storage (67). The clinical relevance of this storage “lesion” to predict the efficacy and safety of the transfusion for the recipient is still a matter of controversy but suggests that RBC quality does not remain stable during storage.

It has been assumed that the decrease in transfusion recovery related to storage is due to RBC damages that accumulate after several weeks of storage. Studies performed more than 50 years ago have shown indeed that the extent of the storage lesion increases with storage duration while transfusion recovery decreases accordingly (5, 8, 10, 45). Few studies have directly explored the correlation between *in vitro* markers of storage lesion and *in vivo* recovery. In these studies, three markers (intracellular ATP, deformability, and morphology) have been shown to correlate with transfusion recovery (Box 1). The proportion of RBC removed from circulation (calculated from transfusion recovery) could correspond to the proportion of RBC, damaged during the storage process, which are over a recipient “clearance threshold.” If this assumption is correct, an optimal marker of storage lesion should identify and quantify the subpopulation of RBC that undergoes early premature clearance.

Box 1 **Connecting storage lesion with transfusion recovery.**

**Intracellular ATP**

An inverse correlation between the intracellular content in ATP in the RCC and transfusion recovery has been reported in a number of studies (1, 7, 9, 44). ATP content declines during long-term hypothermic incubation in a non-physiological solution. This is probably at the root of most RBC alterations that accumulate during storage. Intracellular ATP quantification remains, however, difficult to standardize and allows evaluation of the RCC quality at a cell population rather than a single-cell level.

**Morphology**

At least two studies using the ^51^Cr technique have shown that morphological modifications of RBC in a RCC do correlate with transfusion recovery. In the first study, the proportion of RBC with a discoid shape was positively correlated with transfusion recovery (16), while in the second, the morphology index after rejuvenation correlated with recovery (20). An evaluation of RBC morphology thus seems a good potential predictor of transfusion recovery provided that individual RBC shape can be categorized reliably. However, morphology analyses are low-throughput and operator-dependent making them difficult to standardize and implement. New technologies such as imaging flow cytometry may help circumvent this problem. We have recently identified a subpopulation of small spherocytic RBC that appears and expands during storage with wide variations between donors (68). This spherocytic shift could be a relevant marker as it readily identifies a subpopulation of RBC expected to be cleared rapidly after transfusion. However, direct evidence is lacking that small spherocytic RBC are prematurely cleared following transfusion, hence account for all or part of a suboptimal recovery.

**Deformability**

A recent study in patients with thalassemia showed that the increase in hemoglobin following transfusion was inversely correlated to the proportion of “less deformable” RBC in the RCC (57). In this study, a cell flow analyzer (69) was used to measure the elongation index of individual RBC that adheres to a polystyrene slide. Deformability can also be evaluated by measuring an RBC elongation index using ektacytometry (70). Both technologies have shown a decrease in RBC deformability during storage but the cell flow analyzer, although not commercially available has the advantage of measuring individual RBC elongation. In principle, the automated rheoscope and cell analyzer would provide interesting individual cell data on the evolution of RBC during storage.

The spleen has a specific filtering function that operates the clearance of damaged or senescent RBC from the circulation. Knowledge on the spleen filtration process is therefore relevant to understand transfusion recovery.

### Spleen Filtration Capacity

In the splenic circulation, RBCs engage into two parallel pathways, the fast or slow microcirculations (71). In the fast and “closed” microcirculation, RBCs remain in endothelialized pathways and transit from arterioles to the venous sinus lumen through pathways in the perifollicular zone (72). In the slow and “open” microcirculation, RBCs navigate in tortuous microcirculatory beds of the red pulp, devoid of endothelium, before returning to the venous circulation by squeezing through 1- to 2-μm-wide slits between endothelial cells in the wall of sinuses (71, 73). Macrophages account for approximately half the volume of the cords, and their abundance facilitates direct RBC–macrophage interactions (71). The spleen likely contributes through one or more of these mechanisms to the clearance of transfused RBC. Intensity and kinetics of this clearance depend on the proportion of altered RBC in an RCC and on the intensity of the alterations. In physiologic conditions, the spleen can process at least 20 ml of RBC per day. It is conceivable that its filtration capacity might be overwhelmed by the amount of damaged RBC transfused, potentially leaving in circulation RBC that should normally be removed.

### Spleen Filtration Threshold

The spleen-specific filtration process can trigger the clearance of senescent or altered RBC based on the sensing of surface modifications, mechanical alterations, or a combination of both. Inter-endothelial slits in the spleen exert a stringent challenge on RBC and retain least deformable ones (74, 75). Macrophages sense the shape and altered deformability of RBC and phagocytize them (76). In hereditary spherocytosis, morphology and deformability of RBC are linked (77), surface area-to-volume ratio being the main major determinant of RBC ability to cross narrow inter-endothelial slits in the spleen (74, 78). *Ex vivo* experiments with human spleens have confirmed the correlation between RBC retention in the spleen and the loss in projected surface area (79). Retention was almost complete when more than 17.5% of surface area had been lost. There is therefore a “splenic clearance threshold” that senses biomechanical and morphological changes of RBC which has also been determined by modeling *in silico* (75). Deformability and morphology of transfused RBC are expected to be very important determinants of transfusion recovery.

## Conclusion

Recovery of autologous RBC in healthy non-anemic recipients using ^51^Cr labeling, 24 h after transfusion, is the method usually performed to determine the validity of the RCC preparation/storage processes. When examining transfusion recovery studies, we identified three parameters, namely transfusion volume, labeling protocol and the recipient pathophysiological state that have been under-evaluated and may impact the determination of transfusion recovery. For example, monocytes and macrophages, that possess a limited clearance capacity (80), could be saturated and leave in circulation damaged RBC when a large volume of RBC is transfused. Such an assumption is supported by data showing that transfusion of more than five RCC leads to a decreased deformability of circulating RBC (81). In the case of the labeling protocol, it has been shown that RBC stored for a long period are “primed” and more sensitive to an incubation in medium at 37°C (82) and may react differently when incubated in non-physiological solutions used in certain labeling protocol. Also, the observation that transfusion recovery is reduced in recipients with a splenomegaly (4, 61, 83) strongly suggests that individual characteristics or a pathological condition in the recipient impacts the recovery and survival of transfused RBC, even in absence of alloantibodies. These technical differences between transfusion recovery studies in healthy volunteers and transfusion of an anemic patient in a medical context suggest that available transfusion recovery data may not reflect transfusion efficacy in anemic recipients in some physiopathological conditions. A better understanding of RBC clearance mechanisms is warranted and could be explored by conducting transfusion recovery studies. In doing so, an appropriate experimental design, considering the strengths, weaknesses of the available methods, should be selected. As such, antigen mismatch method appears to offer a number of theoretical advantages over the other methods but would ideally be coupled with a non-radioactive labeling method to evaluate the RBC volume in the recipient.

That some storage lesion markers correlate with transfusion recovery reinforces the potential relevance of these *in vitro* studies which may deliver clinically relevant information. However, in the current conditions of blood collection and processing, this correlation between transfusion recovery and storage lesion remains poorly explored. Identification of a marker that could predict transfusion recovery would be a valuable tool for transfusion medicine and help to bridge the gap between storage lesion and the morbi-mortality studies. Future studies that evaluate transfusion recovery should be designed to include selected storage lesion markers to verify potential correlations. Deformability and morphology, preferably at the individual RBC level, appear as key potential markers since both spleen physiology and historical transfusion recovery data identify them as potentially predictive of transfusion recovery.

*In vitro* studies have shown that marked RBC alterations appear and worsen during storage, but a paradox remains since clinical studies have not found correlations between using RCC stored for a short time (generally less than 7–10 days) and improved clinical outcome. This apparent discrepancy is a source of interrogation in the transfusion community. Clinical studies were appropriately designed to guide transfusion policy. The current conclusion is that there would be no benefit at keeping “fresh blood” for specific situations. The impact of studying storage “lesion” has been questioned as well as the medical relevance of cellular alterations that do not translate into any negative outcome. Is storage “lesion” merely a misnomer, to be replaced advantageously by storage “changes”? This is not so sure yet. Many have argued that clinical studies did not assess the effect of transfusing RCC stored for more than 28 days, while storage lesion studies indicate that the extent of damage rapidly increases after 4 weeks of storage (84). Furthermore, clinical studies were not designed to assess transfusion efficacy and particularly the influence of storage duration on transfusion recovery. On the other hand, *in vitro* studies of the storage lesion are not often correlated with *in vivo* recovery. *In vitro* studies of the storage lesion and clinical studies deliver complementary information while addressing different questions. Studies that explore both dimensions of knowledge are difficult to implement since their designs differ. Large safety studies collect simple data from many patients while transfusion recovery collects complex repetitive samples, which are analyzed using relatively sophisticated methods. The way forward is probably to set-up ancillary recovery studies in the context of large safety trials.

## Author Contributions

All the authors listed have made a contribution to the work and approved it for publication.

## Conflict of Interest Statement

PA and PB have a sponsored research agreement with Zimmer Biomet. The remaining author declares that the research was conducted in the absence of any commercial or financial relationships that could be construed as a potential conflict of interest.
